# LC–MS/MS and GC–MS profiling as well as the antimicrobial effect of leaves of selected Yucca species introduced to Egypt

**DOI:** 10.1038/s41598-020-74440-y

**Published:** 2020-10-20

**Authors:** Abeer M. El Sayed, Samar M. Basam, El-Moataz bellah A. El-Naggar, Hanan S. Marzouk, Seham El-Hawary

**Affiliations:** 1 Department of Pharmacognosy, Faculty of Pharmacy, Cairo University, Kasr El Aini, P.O. Box 11562, Cairo, Egypt; 2 Department of Pharmacognosy, Pharos University in Alexandria, Alexandria, Egypt; 3 Department of Pharmacognosy, Faculty of Pharmacy, Damanhour University, Damanhour, Egypt

**Keywords:** Plant sciences, Chemistry

## Abstract

Few studies thoroughly investigated different Yucca species introduced to Egypt. As a part of our ongoing investigation of the Yucca species; *Yucca aloifolia* and its variety *Yucca aloifolia variegata*, *Yucca filamentosa*, and *Yucca elephantipes* (Asparagaceae) were extensively subjected to phytochemical and antimicrobial investigation. Yucca species cultivated in Egypt showed no antimicrobial effect. GC/MS of the lipoid contents *of Y. aloifolia variegata* was carried out. Twenty-six fatty acids were identified. Saturated fatty acids established almost twice the unsaturated ones and constituted 64.64% of which palmitic acid and palmitoleic acid signifying 58.28% and 30.98%, respectively. Hydrocarbons were 21 constituting 39.64% of the unsaponifiable fraction. Only three sterols 42.36% were detected, major was *γ*-sitosterol. LC–MS/MS comparison of the 4 plant extracts imply that *Y.aloifolia variegata* L extract was the richest, which was apparent through its superior biological activity. LC–MS/MS analysis of the total alcoholic extract (Alc) of the leaves of *Y.aloifolia variegata* L. was performed using MS-techniques at different voltages; equal to 35 and 135 eV. Negative and positive-ion modes analyses at low fragmentation energy allowed the tentative identification of 41 and 34 compounds, respectively. The LC–ESI–MS/MS analysis in the positive mode proved to be better in the identification of saponins.

## Introduction

Yucca is a genus of woody perennial shrubs and trees. Its species are notable for their rosettes of evergreen, numerous sword-shaped leaves which are more or less ensiform^1^. Genus Yucca has been estimated to conservatively comprise 35–40 species within its native range from Central America northward to southernmost Canada. However, the species *Yucca aloifolia* L. (Spanish bayonet) grows in Southeastern USA. *Y.elephantipes* (Giant Yucca) is often planted for landscape purposes in urban areas^2^.

Apart from being a source of a wide range of utilitarian products, this genus has found a high reputation in folk medicine. The plant extracts were used to soothe joint pain, bleeding, urethral, and prostate inflammations. Brewed leaves were used for common ailments like psoriasis, dandruff, hair loss, and skin sores. The roots were crushed to make a poultice for wound healing and to cure gonorrhea and rheumatism. Skin emollient, soporific and anti-diabetic agents are other reported uses^2^.

Steroidal saponins, both spirostanol and furostanol type have been isolated from different *Yucca* species^3,4^. Methanolic extract of the leaves of *Y. aloifolia* showed the presence of alkaloids, tannins, steroids, saponins, and flavonoids^5^.

GC–MS analysis offers influence measurements of reproducibility, dynamic range, and universal mass spectral library for compounds with small molecular weight^6,7^. On the other hand, LC–MS covers a large array of compounds predominant as secondary metabolites such as terpenoids and phenolics^8^. Lately, these two methods were equally adapted to allow both inclusive impression and full analysis of critical components of plants^9–11^.

Liquid chromatography coupled to mass spectrometry (LC/MS) is an important analytical technology e.g. metabolomics experiments^12^. LC–MS-based approaches are expected to be of particular importance in plants, owing to the highly rich biochemistry of plants, which covers many semi-polar compounds, including key secondary metabolite groups, which can best be separated and detected by LC–MS approaches^13^. LC–MS/MS is a comprehensive technique; elution order on liquid chromatography column plays a role in the identification of structural isomers e.g. discriminating hexoses. Many saponins existed as isomers of the same molecular weight, suggesting only different sugar or site of glycosylation.

Reviewing the current literature numerous biological activities were reported for different *Yucca* species such as anticoccidial activity^14^. Cheeke et al*.*^15^ proved that *Yucca* polyphenolics may have several roles in anti-inflammatory and anti-arthritic activities. *Y. filamentosa* extracts displayed specific growth inhibitory activity against *Saccharomyces cerevisiae*^16^. Antimicrobial constituents of *Y. shidigera* represented by three butanol-extractable 5*β*-spirostan-3*β*-ol saponins were investigated using *Bacillus pasteurii* and *Saccharomyces cerevisiae* as test prokaryotic and eukaryotic organisms, respectively^17^.

Very few studies were done to investigate different *Yucca* species introduced to Egypt. Therefore, this study aims at investigating the chemical composition and the biological activities of three yucca species cultivated in Egypt; *Yucca aloifolia* and its variety *Y.aloifolia variegata*, *Y. filamentosa* and *Y.elephantipes*.

## Results and discussion

### Antimicrobial effect

Screening of antimicrobial activity was done using the Disc Diffusion Method^18^. Inhibition zones were measured in mm, for the extracts, the standard antibiotics, and the negative control. Results of screening of antimicrobial activity were compared with those obtained by standard antibiotic ciprofloxacin and standard antifungal clotrimazole (Table 1).

**Table 1:** Screening of the antimicrobial activity of four Yucca species; *Y. aloifolia*, *Y.aloifolia variegata* L., *Y. filamentosa* and *Y. elephantipes* using agar diffusion technique, measured inhibition zones (mm).

Sample	*S. aureus*				*B. subtilis*				*P. aeruginosa*				*E. coli*				*C. albicans*			
	C	WE	C	ALE	C	WE	C	ALE	C	WE	C	ALE	C	WE	C	ALE	C	WE	C	ALE
*Y. filamentosa* L. (60;99 mg/ml)^a^	8	8	8	8	8	8	10	12	8	8	8	9	8	8	8	8	8	8	12	13
*Y. aloifolia variegata* L. (30;75 mg/ml)^a^	8	8	8	8	8	8	10	13	8	8	8	8	8	8	8	8	8	8	12	14
*Y. elephantipes* Regel (25;125 mg/ml)^a^	8	8	8	8	8	8	10	10	8	8	8	10	8	8	8	8	8	8	12	14
*Y. aloifolia* (25;75 mg/ml)^a^	8	8	8	8	8	8	10	10	8	8	8	11	8	8	8	8	8	8	12	15
Ciprofloxacin	9	30	9	30	9	30	9	30	9	30	9	30	9	30	9	30	–	–	–	–
Clotrimazole	–	–	–	–	–	–	–	–	–	–	–	–	–	–	–	–	10	17	10	17

C = control (solvent only); WE. = water extract; ALE = alcoholic extract.

^a^Sample concentration used first for the water and the alcoholic extract respectively.

Despite the reports of antimicrobial activities of different species of *Yucca* in literature, the four investigated extracts showed no antimicrobial activities against the tested organisms. Both the water and the alcoholic extract showed no considerable inhibition zones. This may lead to the conclusion that either the species cultivated in Egypt has no antimicrobial constituents or that these constituents exist in the non-polar fraction of the extract. Moreover, these results may be explained by the ineffectiveness of the tested extracts in the given doses upon the tested microorganisms.

Depending on the results achieved from previously reported biological examination^19^, *Y. aloifolia variegata* L. has deemed the most active species and was chosen for further phytochemical investigation. Thus the extraction and phytochemical investigation of this plant is discussed hereafter.

### Investigation of lipid contents

The *n*-hexane fraction of *Y. aloifolia variegata* L. was saponified, and both the saponifiable and the unsaponifiable fractions were obtained and subjected to GC–MS analysis. The results of the analysis are shown in Tables 2 and 3, and Figure S1.

**Table 2:** Results of GC–MS analysis of the fatty acid methyl esters (FAME) of the saponifiable fraction of the dried leaves of *Y.aloifolia variegata.*

Peak	Fatty acids corresponding to identified FAME	Retention time (Rt) in min	Relative retention time (RRt)^a^	Percentage of each component
1	Caproic acid (C6) "S"	3.841	0.178	4.717
2	Caprylic acid (C8) "S"	7.225	0.335	0.002
3	Capric acid (C10) "S"	10.09	0.468	0.005
4	Undecanoic acid (C11) "S"	12.024	0.558	0.005
5	Lauric acid (C12) "S"	14.021	0.651	0.311
6	Tridecanoic acid (C13) "S"	15.657	0.727	0.019
7	Myristic acid (C14) "S"	17.39	0.807	0.048
8	*Cis*-10-pentadecenoic (C15) "U"	19.292	0.896	0.010
9	Pentadecanoic acid (C 15) "S"	19.32	0.897	0.021
10	Palmitoleic acid (C16) "U"	21.085	0.979	30.989
11	Palmitic acid (C16) "S"	21.54	1.000	58.280
12	*Cis*-10-Heptadecenoic acid (C17) "U"	23.678	1.099	0.001
13	Heptadecanoic acid (C17) "S"	24.009	1.115	0.129
14	Gama-linoleic acid (C 18) "U"	25.241	1.172	0.022
15	Linolenic acid (C 18) "U"	25.98	1.206	0.152
16	Oleic acid (C 18) "U"	26.124	1.213	0.950
17	Elaidic acid (C18) "U"	26.185	1.216	0.075
18	Stearic acid (C18) "S"	26.801	1.244	0.298
19	*Cis*-5,8,11,14,17-Eicosapentaenoic acid (C20) "U"	31.122	1.445	0.157
20	Arachidic acid (C20) "S"	33.162	1.540	0.277
21	Heneicosanoic acid (C21) "S"	36.59	1.699	0.065
22	*Cis*-4,7,10,13,16,19-Docosahexaenoic acid (C22) "U"	37.542	1.743	0.151
23	Behenic acid (C22) "S"	39.606	1.839	0.205
24	Tricosanoic acid (C23) "S"	42.174	1.958	0.148
25	Nervonic acid (C24) "U"	43.946	2.040	0.002
26	Lignoceric acid (C24) "S"	44.457	2.064	0.113
	% Saturated fatty acids (SFA)			64.643
	% Unsaturated fatty acids (USFA)			32.509
	% Total fatty acids			97.152

^a^RRt: retention time relative to palmitic acid.

**Table 3:** Results of GC–MS analysis of the unsaponifiable fraction of the *n*-hexane extract of dried leaves of *Y. aloifolia variegata* L*.*

Peak	Identified components	Retention time (R_t_) in min	Relative retention time (RRt)^a^	Percentage of each component
1	Dodecamethylcyclohexasiloxane	18.945	0.342	0.10
2	Tetradecamethylcycloheptasiloxane	23.265	0.420	0.07
3	E-5-octadecene	25.617	0.462	0.28
4	Hexadecamethylcyclooctasiloxane	27.145	0.490	0.070
5	3-Dimethyl-t-butylsiloxy-2,4-dimethylpentan-1-ol	27.968	0.504	0.110
6	1-Octadecene	29.904	0.539	0.63
7	Neophytadiene	30.837	0.556	0.45
8	6,10,14-Trimethyl-2-pentadecanone	31.050	0.560	0.99
9	*Cis*-Bicyclo[10.8.0]eicosane	31.703	0.572	0.29
10	*n*-Eicosane	32.043	0.578	0.26
11	Eicosamethylcyclodecasiloxane	33.528	0.605	0.15
12	E-15-Heptadecenal	33.860	0.611	0.62
13	4-(4-Ethylcyclohexyl)-1-pentyl-cyclohexene	34.539	0.623	0.03
14	2,6,10,15-Tetramethylheptadecane	35.787	0.646	0.33
15	Phytol	36.194	0.653	17.64
16	Z-5-Nonadecene	37.476	0.676	0.61
17	1-iodohexadecane	39.267	0.708	0.17
18	(Z)-9-Tricosene	40.837	0.737	0.61
19	*n*-Pentacos-3-ene	42.467	0.766	0.26
20	Di(2-propylpentyl)phthalic acid ester	43.342	0.782	2.58
21	Stigmasterol	53.011	0.956	4.80
22	Gamma-sitosterol	53.724	0.969	19.92
23	9,19-Cyclolanost-24-en-3-ol	54.615	0.985	5.13
24	4-4(Ethylcyclohexyl)-1-pentylcyclohexene	55.438	1.000	25.91
	% Total area identified			82.01

^a^RRt: retention time relative to 4-4(ethylcyclohexyl)-1-pentylcyclohexene.

Selective ion determination was done by automatically comparing retention times and mass spectra of 37 reference fatty acids methyl esters and those detected in the investigated sample. Quantitative determination was completed using peak area measurement. Results are shown in Table 2 and the total ion chromatogram of fatty acids methyl esters is shown in Figure S1.

In the case of unsaponifiable constituents, identification was done by comparison of their retention times and mass spectra to those indexed in the NIST library and the Wiley database. Only compounds of high matching probability were considered^20^.

Results of GC–MS analysis of the saponifiable fraction (Table 2 & Figure S1) illustrated that: A percentage of 97% of the components were identified. A total of 26 compounds of fatty acids were identified. Saturated fatty acids 64.64% represented double the concentration of unsaturated fatty acids which constituted 32.50%. Palmitic acid and palmitoleic acid represented 58.28% and 30.98% respectively as the major makeup of saturated fatty acids. This is the first and only report of fatty acid components of the hexane fraction of the leaves of *Y. aloifolia variegata* L.

GC–MS analysis characterized 82% of the components of the unsaponifiable fraction (Table 3& FigureS1). Twenty-one hydrocarbons represented 39.64% of the composition of the unsaponifiable fraction. Only three sterols were detected and represented 42.36% of the fraction. However, *γ*-sitosterol was the major constituting 19.92% of the unsaponifiable fraction.

The *n-hexane* fraction was rich in sterols, which agrees with the ability of the plant to synthesize compounds having a steroid nucleus since the main components of the investigated plants are steroidal saponins. *β*-sitosterol, stigmasterol, and cholesterol were already reported in another species, *Y. gloriosa*^21^, other hydrocarbons were reported in *Y. reverchonii*^22^. This is the first study to report the unsaponifiable hydrocarbon and sterol components of *Y.aloifolia variegata* L.

### Qualitative determination of compounds in the total extract of *Y. aloifolia variegata* L. using LC-ESI–MS analysis

LC–MS-based approaches are expected to be of particular importance in plants, owing to the highly rich biochemistry of plants, which covers many semi-polar compounds, including key secondary metabolite groups, which can best be separated and detected by LC–MS approaches^13^. To evaluate the effectiveness of different LC–MS techniques and instruments, analysis in positive mode was done twice using different instruments. The first analysis was done using a Triple quad which offered good results in detection of saponins in the four Yucca extracts. To obtain more decisive and accurate results the analysis was repeated using the more advanced higher resolution Triple TOF, both in positive and negative modes. The results obtained show how the choice of instrument and conditions influences analysis results.

#### ESI–MS for analysis of saponin glycosides

From the analytical perspective, it is clear that steroidal saponins are not detectable by HPLC–UV analysis due to the lack of a strong chromophore and that HPLC analysis of all compounds requires gradient elution. Mass spectrometry represents an effective detection method, and also improvement in selectivity and specificity can be raised by using tandem mass spectrometry. LC–MS is selective and sensitive enough to carry out the analysis of saponins^23,24^.

Electrospray ionization in the positive as well as the negative ion mode, especially in combination with MS–MS techniques, has quickly become the method of choice for sugar sequence analysis. The methodology has the advantage of requiring only minute amounts of substance (< 1 mg) and affords a direct analysis of both purified materials and mixtures without the necessity of derivatization^25^.

Based on these observations, a preliminary analysis of the extract obtained by *Y*. *aloifolia variegata* L. leaves was performed by LC–ESI–MS to detect the presence of saponins in this part of the plant (Tables 4–7 & Figures S2, 3).

**Table 4:** LC–MS/MS data-positive ion mode for compounds identified in the first analysis of *Y. aloifolia variegata* L. leaves total extract with electrospray ionization at rather high fragmentation energy; equal to 135 eV.

Peak no.	Rt	Formula	Ion	MS/MS	Identification
Y1	18.48	C_39_H_64_O_13_	[M + H]^+^	741.0, 579.1, 417.1	Spirostan-3-ol-3-*O*-[*β*-d-galactopyranosyl-(1 → 4)-*β*-d-glucopyranoside
Y2	22.4	C_7_H_6_O_5_	[M + H]^+^	171.0	Gallic acid
Y3	22.92	C_39_H_64_O_13_	[M]^+^	741.0, 595.1, 433.1, 415.0,	Spirostan-diol- rhamnosyl hexosyl
Y4	25.21	C_33_H_52_O_8_	[M + H]^+^	579.1, 431	Hecogenin-rhamnoside
Y5	28.44	C_39_H_61_O_13_	[M + H]^+^	741.1, 579.1, 417.1	(3*β*, 5*α*, 25*R*)-Spirostan-3-ol-3-*O*-[*β*-d-glucopyranosyl-(1 → 2)-*β*-d-glucopyranoside]
Y6	28.91	C_39_H_64_O_13_	[M + H]^+^	741.1, 579.1, 417.1	(3*β*, 5*β*, 25*R*)-Spirostan-3-ol-3-*O*-[*β*-d-glucopyranosyl-(1 → 2)-*β*-d-glucopyranoside]
Y7	29.44	C_39_H_64_O_13_	[M + H]^+^	741.1, 579.1, 417.1	(3*β*, 5*α*, 25*S*)-Spirostan-3-ol-3-*O*-[*β*-d-glucopyranosyl-(1 → 2)-*β*-d-glucopyranoside]
Y8	30.19	C_39_H_64_O_13_	[M + H]^+^	741.1, 579.1, 417.1	(3*β*, 5*β*, 25*S*)-Spirostan-3-ol-3-*O*-[*β*-d-glucopyranosyl-(1 → 2)-*β*-d-glucopyranoside]
Y9	30.5	C_39_H_64_O_14_	[M + H]^+^	758.1, 579.1, 417.1	25*R*-Spiostan-diol-dihexoside isomer
Y10	30.9	C_39_H_64_O_14_	[M + H]^+^	758.1, 579.1, 417.1	25*S*-Spiostan-diol-dihexoside isomer
Y11	31.9	C_39_H_61_O_13_	[M + H]^+^	741.1, 579.1, 417.1	Spirostanol-dihexoside isomer
Y12	36.4	C_16_H_18_O_9_	[M + H]^+^	354.0	Chlorogenic acid
Y13	40.06	C_27_H_30_O_16_	[M + H]^+^	624.0, 610.9	Rutin or Hesperidin
Y14	41.68	C_9_H_8_O_2_	[M + H]^+^	149.9, 148.9	Cinnamic acid

**Table 5:** LC–ESI–MS negative ion mode analysis of the total alcoholic extract of *Y. aloifolia variegata* L. was performed using electrospray ionization at relatively low fragmentation energy; equal to 35 eV.

Comp	Rt	Formula	Ion	MS/MS	Identification
P1	0.47	C_4_H_6_O_5_	[M-H]^−^	133.0135,115.009, 114.9900, 89.0200	Malic acid
P2	0.49	C_28_H_34_O_15_	[M-H]^−^	610. 2056, 609.1945	Hesperidin
P3	0.501	C_22_H_30_O_13_	[M-H]^−^	489.1477, 341.1138, 179.0572, 147.0313	Caffeic acid monohexoside derivative
P4	0.516	C_6_H_12_O_6_	[M-H]^−^	179.0572	Caffeic acid
P5	0.52	C_16_H_20_O_6_	[M-H]^−^	327.1123, 179.0571, 147.0290	Caffeic acid derivative
P6	0.53	C_10_H_10_O_4_	[M-H]^−^	193.0340	Ferulic or isoferulic acid
P7	0.8	C_16_H_12_O_6_	[M-H]^−^	299.0901, 255.1202, 178.0200	3,5,7-trihydroxy-4′-methoxyflavone
P8	0.812	C_21_H_18_O_12_	[M-H]^−^	461.1678, 389.0100, 385.1501, 315.1100	Kaempferol-3-*O*-Glucuronide
P9	0.97	C_21_H_20_O_12_	[M-H]^−^	463.1751, 301.0250	Quercetin-*O*-hexoside
P10	1.059	C_21_H_20_O_11_	[M-H]^−^	447.1903, 385.1103, 297.0202	Quercitrin
P11	1.31	C_20_H_18_O_10_	[M-H]^−^	417.1502, 415.8400, 302.0011,207,0021	Kaempferol-3-*O*-arabinoside
P12	1.97	C_16_H_18_O_9_	[M-H]^−^	353.1459	Chlorogenic acid
P13	2.2	C_21_H_20_O_10_	[M-H]^−^	431.1631, 413.3001, 397.1201,201.0110	Kaempferol-3-*O*-rhamnoside
P14	2.3	–	[M-H]^−^	595.2602, 448.1817, 431.1930	Saponin cinnamoyl derivative
P15	3.14	C_27_H_32_O_15_	[M-H]^−^	595.2604, 441.1990	Neoeriocitrin
P16	3.25	C_21_H_24_O_9_	[M-H]^−^	419.1364, 337.1521, 294.8212	Dihydroxymethoxy-glucopyranosylstilbene
P17	3.3	C_27_H_30_O_16_	[M-H]^−^	609.1486,449.1601, 301.0412	Rutin
P18	3.77	C_15_H_12_O_6_	[M-H]^−^	287.0516	3,7,3′4′-tetrahydroxyflavanone
P19	3.81	C_33_H_56_O_6_	[M-H]^−^	577.2502, 431.2069	Kaempferol dirhamnoside
P20	3.86	C_27_H_30_O_12_	[M-H]^−^	567.2283, 405. 1753, 179.0515	Dihydrosinapyl caffeoyl hexoside
P21	3.93	C_30_H_26_O_13_	[M-H]^−^	593.1538, 293.1421, 285.0612	Kaempferol-3-*O*-(*p*-coumaroyl)-glucoside
P22	4.1	C_28_H_32_O_16_	[M-H]^−^	623.165,315.0312, 299.0316	Isorhamnetin-3-O-rutinoside
P23	4.16	C_18_H_16_O_8_	[M-H]^−^	359.1372, 285.0421, 257.0316	Rosmarinic acid
P24	4.2	–	[M-H]^−^	577.2495, 341.1110, 179.0535	Caffeic acid-O- hexoside derivative
P25	4.27	C_39_H_61_O_13_	[M-H]^−^	739.7912, 577. 4151	Spirostan-3-ol-glucoside-galactoside
P26	4.77	C_27_H_42_O_4_	[M-H]^−^	429.1700	Hecogenin
P27	4.83	C_20_H_22_O_9_	[M-H]^−^	405.1708, 322.0406, 164.0412	Trihydroxyglucopyranosylstilbene
P28	4.89	–	[M-H]^−^	586.2758, 407.1857, 301.0746	Caffeoyl quercetin derivative
P29	5.13	–	[M-H]^−^	571.2909 , 327.2197	Unknown
P30	5.55	C_15_H_16_O_9_	[M-H]^−^	339.1217, 270.0071, 192.2312	Esculin
P31	6.14	C_39_H_61_O_13_	[M-H]^−^	739.7912, 577, 4150	Spirostan-3-ol-dihexoside
P32	6.26	C_15_H_12_O_5_	[M-H]^−^	271.1552, 211.0151, 185. 0912, 56.0110	Naringenin
P33	6.70	C_15_H_10_O_7_	[M-H]^−^	301.0378, 284.1221, 255.2316	Quercetin
P34	6.77	C_15_H_10_O_6_	[M-H]^−^	285.0396, 257.0512, 229.0516	Luteolin
P35	7.11	C_16_H_14_O_6_	[M-H]^−^	301.0260, 285.9020	Hesperetin
P36	7.24	C_16_H_12_O_5_	[M-H]^−^	283.0617, 177.0812, 171.05,1510	Acacetin
P37	7.45	C_30_H_26_O_12_	[M-H]^−^	577.2699 , 417.1785	Procyanidin B2
P38	8.04	C_15_H_10_O_5_	[M-H]^−^	269.0797, 227.0660, 148.0100	Apigenin
P39	11.40	C_15_H_10_O_6_	[M-H]^−^	285.0772, 239.0312	Kaempferol
P40	13.80	C_27_H_44_O_3_	[M-H]^−^	461.2620	Spirostanol
P41	16.93	C_18_H_32_O_2_	[M-H]^−^	279.2016	Linoleic acid

**Table 6:** LC–MS/MS data-positive ion mode for compounds (P'1-34) identified in analysis of *Y. aloifolia variegata* L. leaves total extract which was performed using electrospray ionization at relatively low fragmentation energy; equal to 35 eV.

Peak	Rt	Formula	Ion	M, MS/MS	Identification
P'1	0.489	C_30_H_26_O_13_	[M + H]^+^	595.1432	Kaempferol-3-*O*-(*p*-coumaroyl)-glucoside
P'2	0.5	C_21_H_20_O_12_	[M + H]^+^	465.1015	Hyperoside
P'3	0.52	C_21_H_28_O_14_	[M + Na]^+^	527.1594, 365.1031, 203.0554, 180.0390	Caffeoyl dihexoside
P'4	0.55	C_7_H_6_O_5_	[M + Na]^+^	193.1101	Gallic acid
P'5	0.62	C_10_H_10_O_4_	[M + H]^+^	195.1240	Ferulic acid
P'6	1.18	C_9_H_8_O_2_	[M + H]^+^	149.0241	Cinnamic acid
P'7	2.118	C_16_H_22_O_9_	[M + H]^+^	359.1725, 242.9480, 197.1160	Sweroside
P'8	2.9186	C_10_H_8_O_2_	[M + H]^+^	161.1307, 119.0806	6-Methylcoumarin
P'9	3.535	C_27_H_30_O_16_	[M + H]^+^	611.1210, 201.0612	Hesperidin
P'10	3.61	C_39_H_64_O_13_	[M + Na]^+^	779.3670, 757.4638, 595.3807, 577.3860, 433.3269, 415.3176	Spirostan-3,12-diol-3-*O*-glucopyranosyl-(1 → 2)-glucopyranosyl
P'11	3.69	C_11_H_16_O_3_	[M + H]^+^	197.1173, 179.1211, 135.1100, 133.0211	Loliolide
P'12	4.29	C_28_H_32_O_16_	[M + H]^+^	625.1670	Isorhamnetin-3-*O*-rutinoside
P'13	4.56	C_33_H_52_O_8_	[M + H]^+^	593.3679, 431.3193, 413.3006	Yucca spirostanoside B1
P'14	6.14	C_9_H_8_O	[M + H]^+^	133.1013, 131.08, 117.0665, 115.0501, 91.0212	Cinnamaldehyde
P'15	6.2–6.27	C_39_H_64_O_13_	[M + H]^+^	741.4404, 579.3858, 417.1661	7 spirostanol dihexoside isomers
P'16	6.24	C_45_H_74_O_18_	[M + H]^+^	903.4864, 741.4528, 579.3898, 417.3521	Spirostanol trihexoside
P'17	6.818	C_39_H_62_O_14_	[M + H]^+^	755.337,593.3220	5*β*-Spirostan-3*β*,12*β*-diol 3-*O*-*β*-d-glucopyranosyl-(1 → 2)-*O*-*β*-d-glucopyranoside
P'18	7.13	C_19_H_24_O_2_	[M + H]^+^	285.0771, 284.7700, 135.0112, 122.0313	Androsta-1,4-diene-3,17-dione
P'19	7.76	C_45_H_74_O_18_	[M + H]^+^	903.4957, 741.4380, 579.3868, 417.3351	Spirostanol trihexoside isomer
P'20	7.937	C_16_H_12_O_5_	[M + H]^+^	286.8812, 147.0110	4′,5-dihydroxy-7-methoxyflavone
P'21	8.6	C_15_H_10_O_6_	[M + H]^+^	287.1269	Luteolin
P'22	8.96	C_27_H_32_O_14_	[M + H]^+^	581.1996, 419.3506, 273.2141	Naringin
P'23	9.13	C_16_H_12_O_5_	[M + H]^+^	285.1111	Acacetin
P'24	9.56	C_27_H_42_O_4_	[M + H]^+^	431.3140, 413.3011,395.2912, 299.2416	Hecogenin
P'25	10.70277	C_15_H_10_O_7_	[M + H]^+^	303.2765	Quercetin
P'26	9.304517	C_15_H_10_O_6_	[M + H]^+^	287.1490	Kaempferol
P'27	9.56	C_15_H_10_O_5_	[M + H]^+^	271.1528	Apigenin
P'28	13.94	C_27_H_44_O_3_	[M + Na]^+^	438.2897, 377.1358, 253.1430	Spirostanol
P'29	16.1	C_20_H_40_O	[M + H]^+^	297.2404, 281.0612, 248.9901	Phytol
P'30	17.23	C_38_H_60_O_12_	[M + Na]^+^	747.2970, 433.1765	Yucca spirostanoside B2
P'31	19.16	C_27_H_44_O_3_	[M + H]+	417.3366	Sarsasapogenin
P'32	19.8897	C_27_H_42_O_3_	[M + H]+	415.3205, 283,2412, 271.2016	Diosgenin
P'33	20.09	C_27_H_46_O	[M-H_2_O + H]^+^	369.3361, 351.3355, 123.1190	Cholesterol
P'34	25.594	C_29_H_52_O	[M-H_2_O + H]^+^	399.3626, 109.0612, 57.8616	Stigmastanol

**Table 7:** Comparison in the identified compounds in the alcoholic extracts of the *Y. aloifolia variegata, Y. aloifolia, Y. filamentosa and Y.elephantipes.*using LC–MS/MS.

Peak no.	Rt	Identification	*Y. aloifolia variegata*	*Y. aloifolia*	*Y. filamentosa*	*Y.elephantipes*
1	18.48	Spirostan-3-ol-3-*O*-[*β*-d-galactopyranosyl-(1 → 4)-*β*-d-glucopyranoside	++	+	+	+
2	22.40	Gallic acid	++	+	−	+
3	22.92	Spirostan-diol- rhamnosyl hexosyl	++	+	−	−
4	25.21	Hecogenin-rhamnoside	++	+	−	+
5	28.44	(3*β*, 5*α*, 25*R*)-Spirostan-3-ol-3-*O*-[*β*-d-glucopyranosyl-(1 → 2)-*β*-d-glucopyranoside]	++	+	−	+
6	28.91	(3*β*, 5*β*, 25*R*)-Spirostan-3-ol-3-*O*-[*β*-d-glucopyranosyl-(1 → 2)-*β*-d-glucopyranoside]	++	+	−	+
7	29.44	(3*β*, 5*α*, 25*S*)-Spirostan-3-ol-3-*O*-[*β*-d-glucopyranosyl-(1 → 2)-*β*-d-glucopyranoside]	++	+	−	+
8	30.19	(3*β*, 5*β*, 25*S*)-Spirostan-3-ol-3-*O*-[*β*-d-glucopyranosyl-(1 → 2)-*β*-d-glucopyranoside]	++	+	−	+
9	30.50	25*R*-Spiostan-diol-dihexoside isomer	++	+	−	+
10	30.90	25*S*-Spiostan-diol-dihexoside isomer	++	+	−	+
11	31.90	Spirostanol-dihexoside isomer	++	+	−	−
12	36.40	Chlorogenic acid	++	+	+	+
13	40.06	Rutin or Hesperidin	++	+	+	−
14	41.68	Cinnamic acid	++	+	+	+

(++) present in high concentration, (+) present in lower concentration, (−) absent.

Analysis of the total alcoholic extract of *Y. aloifolia variegata* L. was first performed using electrospray ionization at rather high fragmentation energy; equal to 135 eV.

This relatively high energy harmed the analysis and lead to the extensive fragmentation of compounds present in the extract. This leads to the identification of only the compounds present in high concentrations.

Mass spectra obtained from the analysis allowed the tentative identification of 14 compounds (Y1-14) presented in (Table 4& Figure S4). Saponins comprised the majority of the identified compounds; precisely 10 saponin peaks were identified. Identified saponins belonged to three groups, as revealed by their aglycone peaks.

Spirostanol saponins were identified based on the presence of the aglycone peak at m/z 417, corresponding to [aglycone + H], suggested a saturated monohydroxy spirostanol skeleton^26–28^. In some cases, the aglycone peak appeared at m/z 415, corresponding to either [aglycone + H] or [aglycone + H-H_2_O], which indicates either the existence of unsaturation in the aglycone skeleton or the existence of another hydroxyl group if preceded by a peak at m/z 433^29^. Where the 18 Da difference between the two peaks accounts for the loss of one water molecule at the extra hydroxyl and formation of an unsaturation. Another type of spirostanol saponins is hecogenin which gives aglycone peak at m/z 431. Hecogenin has been reported in several species of *Yucca*.

Substituent sugars were observed as neutral atom losses from mass fragments; these were identified as hexoses and deoxyhexoses. According to literature, hexoses attached to yucca saponins include *β*-d-glucopyranosides and *β*-d-galactopyranosides. On the other hand, the only reported deoxyhexose in yucca is *α*-L-rhamnopyranose. Compounds identified as saponins were Y1, Y3-Y11.

Compound Y1 showed a typical mass spectrum of a monohydroxy unsaturated spirostanol dihexoside. The mass fragmentation showed pseudo molecular ion peak [M + H]^+^ at m/z 741, followed by a peak at m/z 579 due to the loss of hexose moiety, thus corresponding to [M + H-162]^+^ and then the aglycone peak at m/z 417; similar compounds were previously reported in genus *Yucca*; either as diglucopyranosyl derivatives or glucopyranosyl-galactopyranosyl derivatives. The compound was identified as spirostanol-dihexoside.

Compound Y3 showed peaks at m/z 433 and m/z 415, indicating it is a dihydroxy spirostanol. The peak at m/z 741 was the molecular ion peak [M]^+^, followed by a peak at m/z 595, due to the loss of a deoxyhexose unit, thus corresponding to [M-146]^+^, followed by a peak at 433 resulting from the loss of hexose unit, and finally, a peak at 415 due to loss of one water molecule. The compound was identified as spirostan-diol-rhamnosyl hexosyl. Since the galactosides elute at shorter retention times than glucosides, and since rhamnoside on the contrary elutes at longer retention times, therefore, compound Y3 is expected to be Spirostan-diol- rhamnosyl galactoside. As the combination of the galactosyl with rhamnosyl moieties may be the reason for the shorter retention time than peaks Y9 and Y10.

Compound Y4 showed aglycone peak at m/z 431, corresponding to [aglycone + H]^+^, indicating a 12-oxo-spirostanol genin, most probably, hecogenin. The pseudomolecular ion peak appeared at m/z 741, corresponding to [M + H]^+^. The neutral atom loss of 146 Da indicated a rhamnosyl moiety. The compound was identified as hecogenin rhamnoside.

Compounds Y5–Y8 and Y11 appeared as signals at different retention times in the total ion chromatogram. However, after tandem mass analysis, they showed the same mass spectrum as that of Y1. As a result, these compounds were assumed to be isomers Y1. Thus, they were identified as spirostanol-dihexoside isomers.

Although mass spectrometry is not commonly known to discriminate isomers, a combination of MS data with HPLC retention times and elution order can be used integrative for this purpose.

According to the Dictionary of Natural Products, 5 isomers of spirostanol dihexosides are reported to have the same molecular weight 740 amu; Spirostan-3-ol; (3*β*,5*α*,25*R*)-form, 3-*O*-[*β*-d-Galactopyranosyl-(1 → 4)-*β*-d-glucopyranoside] and the four diastereomers of Spirostan-3-ol-3-*O*-[*β*-d-glucopyranosyl-(1 → 2)-*β*-d-glucopyranoside], namely; (3*β*,5*α*,25*R*), (3*β*,5*α*,25*S*), (3*β*,5*β*,25*R*) and (3*β*,5*β*,25*S*). Compound Y1 at retention time 18.48 can be assigned to compound Spirostan-3-ol-3-*O*-[*β*-d-galactopyranosyl-(1 → 4)-*β*-d-glucopyranoside, because galactosides are reported to elute before glucosides^30^.

Unfortunately, the discrimination of the four 5*α*/*β*, 25 *R*/*S* diastereomers of spirostanol-3-*O*-diglucoside are not possible using C18 RP^31^. Proper separation of 25*R*/*S*-spirostanol saponin diastereomer was achieved by using the C30 RP column, which leads to the observation that the retention time of 25*R*-spirostanol saponins was always shorter than that of 25*S* ones^32^. However, no more data could be achieved to propose the possible exact structure of the sixth isomer that elutes later at retention time 31.9 min.

Compounds Y9 and Y10 also appeared as two consecutive signals at different retention times in the total ion chromatogram. However, they showed the same mass spectrum. The spectrum showed a molecular ion peak [M]^+^ at m/z 758, followed by a peak at m/z 579, due to the simultaneous loss of a hydroxyl unit and a hexose unit, thus corresponding to [M-17-162]^+^. The aglycone peak [M-17-162-162]^+^ appeared at m/z 417 after the loss of another hexose unit. Compounds were identified as spirostan-diol-dihexoside isomers. These may be suggested to be 25*R*–diglucoside isomer followed by the 25*S* isomer. According to the Dictionary of Natural Products, reported spirostan-diols are Spirostane-3,12-diol; (3*β*,5*α*,12*β*,25*R*)-form and (3*β*,5*β*,12*β*,25*R*)-form, Spirostane-2,3-diol; (2*α*,3*β*,5*α*,25*R*)-form, (2*β*,3*β*,5*β*,25*S*)-form and (2*α*,3*β*,5*α*,25*S*)-form, and Spirostane-3,6-diol; (3*β*,5*α*,6*α*,25*R*)-form. Compounds Y9 and Y10 can have the second hydroxyl at any of these positions; C2, C12, or C6. Unfortunately, LC–MS/MS cannot distinguish them in the absence of standards.

Despite the prior identification of phenolic acids and flavonoids in the extract, only 3 phenolic acids and one flavonoid could be thoroughly identified in this analysis. This may be traced back to the high fragmentation energy used, which leads to the extensive fragmentation of most phenolic acids and flavonoids^33^. Characteristic fragment ions of flavonoids such as; 273, 255, 283, 181,179, 151 were detected through-out the total ion chromatogram^30^. However, in most cases, no proper spectrum could be achieved to aid the identification. The exception was the 3 phenolic acids and the flavonoid identified, most probably due to their presence in higher concentrations, which allowed the survival of some of their characteristic ions.

Compound Y2 was identified as gallic acid due to its pseudo molecular ion peak [M + H]^+^ at m/z 171^34^. Compound Y12 was identified as chlorogenic acid due to its molecular ion peak [M]^+^ at m/z 354. Compound Y14 was identified as cinnamic acid due to its pseudo molecular ion peak [M + H]^+^ at m/z 149^35^.

Compound Y13 was identified as either hesperidin or rutin, due to pseudo molecular ion peak [M + H + Na]^+^ at m/z 624 and ion peak [M + H]^+^ at 611^36,37^. The results of this analysis indicated that the positive ion mode ESI proved good in the analysis of saponins than phenolics.

Consequently, it was deemed necessary to carry out another analysis at lower fragmentation energy, in both positive and negative modes for better investigation of the chemical composition of the extract of *Y.aloifolia variegata* L.

### Investigation of the chemical composition of the total alcoholic extract of *Yucca aloifolia variegata* L. using LC–ESI–MS analysis

Analysis of the total alcoholic extract of *Y. aloifolia variegata* L. was performed using electrospray ionization at relatively low fragmentation energy; equal to 35 eV.

- Analysis in the Negative-ion Mode

This relatively low energy had a good impact on the analysis and leads to better identification of flavonoids and phenolic acids and their glycosides. Mass spectra obtained from the analysis allowed the tentative identification of 41 compounds (P1-41) (Table 5 & Figure S5). However, a total of 5332 unknown peaks were detected, reflecting the highly complex nature of the extract.

Identified compounds can be classified into phenolic compounds and their derivatives, represented by compounds P3-P6, P12, P20, P23, P24, and P37, 21 flavonoids, represented by compounds P2, P7-P11, P13, P15, P17-22, P28, P32-34, P36, P38, and P39. The analysis also leads to the tentative identification of 5 saponin derivatives, represented by compounds P14, P25, P26, P31, and P40. Other compounds, such as coumarins and stilbene derivatives could be identified.

The identification was done by comparison of their exact masses and fragmentation patterns to data recorded in the in-house database of 57357 proteomics laboratory. Other compounds were identified by this study through comparison of their masses and fragmentation patterns to reported literature.

#### Identification of Phenolic acids

Identified phenolic acids include low molecular weight phenolic acids, in addition to their glycosides.

Compounds P1 aliphatic and P5 were identified by the in-house database as malic acid and caffeic acid; with pseudomolecular ion peaks [M-H]^−^ at m/z 133.0135 and 179.0577, respectively^38,39^.

Compound P3 showed a pseudomolecular ion peak [M-H]^−^ at m/z 489.1477, followed by a peak at 341.1138, corresponding to [M-H-148]^−^ due to loss of an unknown moiety^40^ which might be cinnamic acid unit^41^. The peak at m/z 341.1138 is characteristic of caffeic-monoglycoside^42^ followed by a peak at m/z 179.0572, corresponding to [M-H-148-162]^−^, due to the loss of one hexose unit attached to the caffeic acid. Peak at m/z 147.0313, corresponds to [M-H-148-162-32]^−^, due to loss of O_2_. Compound P3 was identified as a caffeic acid monoglycoside derivative.

Compound P5 showed a pseudomolecular ion peak [M-H]^−^ at m/z 327.1123, followed by a peak, corresponding to [M-H-148]^−^ due to loss of unknown moiety of mass 148 amu, leaving a peak at m/z 179.0572 corresponding to caffeic acid^43^, followed by a base peak at m/z 147.0295, corresponding to cinnamic acid. Compound P6 was identified as caffeic acid derivative^44^.

Compound P12 showed a pseudo molecular ion peak [M-H]^−^ at m/z 353.1459 and was identified as Chlorogenic acid^45^, previously identified in the first analysis.

Compound P20 showed a pseudo molecular ion peak [M-H]^−^ at m/z 567.2283, followed by a peak at m/z 405.1753, due to loss of one hexose unit, corresponding to [M-H-162]^−^, and finally a peak at m/z 179.0515 due to caffeic acid nucleus. Compound was identified as dihydrosinapylcaffeoylhexoside^46^.

Compound P23 showed a pseudo molecular ion peak [M-H]- at m/z 359.1372 and was identified as rosmarinic acid^47^.

Compounds P24 showed a pseudo molecular ion peak [M-H]^−^ at m/z 577.2495, followed by a peak at m/z 341.1110. A base peak at m/z 179.0535 was observed, corresponding to the caffeic acid nucleus, formed after the loss of hexose unit form the previous fragment. The compound was identified as a caffeoyl hexose derivative^48^.

Compound P37 showed pseudo molecular ion peak [M-H]^−^ at m/z 577.26997, followed by a peak at m/z 417.1785, due to loss of 18 amu from 435 fragments^49^. The compound was identified as E-catechin dimer Procaynidin B2 previously reported in *Y. elephantipes* flowers^50^.

According to this analysis, caffeic acid and more than one derivative are found in the extracts. Caffeic acid was also identified by HPLC in the extract using an external standard.

#### Identification of Flavonoids

Identified flavonoids can be classified into:

*Flavones and flavone glycosides*

Compounds P34, P36, and P38 showed pseudo molecular ion peaks [M-H]^−^ at m/z 285.0396, 283.061, and 269.0797, respectively. These compounds were identified as luteolin, acacetin, and apigenin.

*Flavanones and flavanone glycosides*

Compounds P2, P35, and P32 showed pseudo molecular ion peaks [M-H]^−^ at m/z 609.1945, 301.026, and 271.1552, respectively. These compounds were identified by comparison of their exact masses and fragmentation patterns and were deduced to be hesperidin, its aglycone hesperetin, and naringenin.

Compound P15 showed pseudo molecular ion peaks [M-H]^−^ at m/z 595.2604 and was identified by comparison of its exact mass as Neoeriocitrin.

*Flavonols and Flavonol glycosides*

Quercetin and its glycosides were identified in the extract. Compound P33 showed a pseudomolecular ion peak [M-H]^−^ at m/z 301.03378. Compounds P9, P10 showed pseudo molecular ion peaks [M-H]^−^ at m/z 463.1751, 447.1903, and were identified as quercetin-*O*-hexoside and quercetin.

Compound P28 showed a pseudomolecular ion peak [M-H]-at m/z 586.2758, followed by a peak at m/z 407.1857, corresponding to [M-H-179]^−^, due to loss of caffeoyl moiety. The peak at m/z 301.0746 indicates the quercetin nucleus. The compound was identified as a derivative of caffeoyl-quercetin.

Kaempferol was identified at peak P39, showing a pseudomolecular ion peak [M-H]^−^ at m/z 285.0772. Compounds P8, P11, P13, and P21 showed pseudo molecular ion peaks [M-H]^−^ at m/z 461.1678, 417.1502, 431.1631, and 593.1538, identified by the in-house database as kaempferol-3-*O*-glucuronide, kaempferol-3-*O*-arabinoside, kaempferol-3-*O*-rhamnoside and kaempferol-3-*O*-(*p*-coumaroyl)-glucoside, respectively.

Compound P24 showed pseudo molecular ion peak [M-H]^−^ at m/z 577.2502, followed by a peak at m/z 431.2069. The difference between fragments masses was 146 Da indicating the loss of a rhamnosyl moiety. The compound was identified as Kaempferol-dirhamnoside, already identified by HPLC analysis^51^.

Compounds P17, P22 showed pseudo molecular ion peaks [M-H]^−^ at m/z 609.1486 and 623.165, respectively, and were identified by the in-house database as rutin and isorhamnetin-3-*O*-rutinoside.

#### Identified saponins

Compound P31 representing the major peak at 6.14 min showed pseudo molecular ion peaks [M-H]^−^ at m/z 739.79 assigned to spirostanol-3-ol-dihexoside, previously identified as more than one isomer in the first analysis.

Compound P25 at 4.27 min showed pseudo molecular ion peak [M-H]^−^ at m/z 739.79, corresponding to spirostan-3-ol-dihexoside, already identified in the first analysis.

Compound P31 is believed to be the diglucoside while P25 the glucoside-galactoside isomer, due to the order of elution previously explained in the first analysis.

Compound P26 showed pseudo molecular ion peaks [M-H]^−^ at m/z 429.17, characteristic of hecogenin, No further fragmentation was observed. The compound was identified as hecogenin.

Compound P40 showed a pseudo molecular ion peak [M-H + HCOOH]^−^ at m/z 461.26 corresponding to the formic adduct of spirostan-3-ol, a major saponin aglycone in *Yucca*.

#### Other identified compounds

Compounds P16 and P27 showed pseudo molecular ion peaks [M-H]^−^ at m/z 419.1364 and 405.1708, respectively. Compounds were identified as dihydroxymethoxy-glucopyranosylstilbene and trihydroxyglucopyranosylstilbene.

Compound P36 showed pseudo molecular ion peak [M-H]^−^ at m/z 339.1217 and was identified as esculin.

Compound P41 showed pseudo molecular ion peak [M-H]^−^ at m/z 279.2 and was identified as linoleic acid.

- Analysis in the Positive-ion Mode

A total of 34 compounds (P'1–34) were identified. However, a total of 3706 unknown peaks were detected, confirming the highly complex nature of the extract (Table 6 & FigureS6).

The analysis in the positive mode proved to be better in the identification of saponins, giving more explanatory fragmentation patterns. However, other classes of compounds were also identified, including phenolic acids, flavonoids, and other compounds of steroidal nature.

#### Identification of saponins

Compound P'10 showed a pseudomolecular ion peak [M + Na]^+^ at m/z 779.3670, followed by a peak at m/z 757.4638, corresponding to [M + H]^+^, followed by a peak at m/z 595.3807, due to the loss of one hexose unit giving [M + H-162]^+^. The loss of a water molecule leads to a peak at m/z 577.3860, corresponding to [M + H-162–18]^+^, followed by the loss of another hexose unit giving a peak of [M + H-162-162]^+^ at m/z 433.3269, and finally the loss of another water molecule giving [M + H-162-18-162-18]^+^ at m/z 415.3176. This pattern confirms the loss of two water molecules due to two hydroxyls and two hexoses. The compound was previously reported as Spirostan-2, 3-diol-3-*O*-galactopyranosyl-(1 → 2)-glucopyranoside^52^.

Compound P'13 showed a pseudomolecular ion [M + H]^+^ at m/z 593.3679, followed by a peak at m/z 431.3193, due to loss of one hexose unit giving fragment ion [M + H-162]^+^, followed by a peak at m/z 413.3006 due to the loss of a water molecule, giving [M + H-162-18]^+^. The compound was previously reported as yucca spirostanoside B1^53^.

Compounds P'15 and P'19 appeared at different retention times in the total ion chromatogram. However, both showed the same mass spectra indicating they are isomers. The spectrum showed a pseudomolecular ion [M + H]^+^ at m/z 903.4864, followed by three successive peaks at m/z 741.4528, 579.3858, and 417.1661, due to the loss of three hexose units giving fragment ions [M + H-162]^+^, [M + H-162-162]^+^and [M + H-162-162-162]^+^. The peak at 417.1661 indicated a spirostanol derivative. Compounds were identified as spirostanol trihexoside isomers. These compounds are reported in the Dictionary of Natural products database as Spirostan-3-ol; (3*β*,5*β*,25*S*)-form,3-*O*-[*β*-d-Glucopyranosyl-(1 → 2)-[*β*-d-glucopyranosyl-(1 → 3)]-*β*-d-glucopyranoside], Spirostan-3-ol; (3*β*,5*β*,25*S*)-form, 3-*O*-[*β*-d-Glucopyranosyl-(1 → 2)-[*β*-d-glucopyranosyl-(1 → 3)]-*β*-d-galactopyranoside], Spirostan-3-ol; (3*β*,5*β*,25*R*)-form, 3-*O*-[*β*-d-Glucopyranosyl-(1 → 2)-[*β*-d-glucopyranosyl-(1 → 3)]-*β*-d-glucopyranoside], Spirostan-3-ol; (3*β*,5*β*,25*R*)-form, 3-*O*-[*β*-d-Glucopyranosyl-(1 → 2)-[*β*-d-glucopyranosyl-(1 → 3)]-*β*-d-galactopyranoside], Furost-20(22)-ene-3,26-diol; (3*β*,5*β*,25R)-form, 3-O-[*β*-d-Glucopyranosyl-(1 → 2)-*β*-d-glucopyranoside], 26-*O*-*β*-d-glucopyranoside.

As previously discussed, galactosides elute before glucosides, so P'15 is assumed to be (3*β*,5*β*,25*S*/R)- Spirostan-3-*O*-[*β*-d-Glucopyranosyl-(1 → 2)-[*β*-d-glucopyranosyl-(1 → 3)]-*β*-d-galactopyranoside] and P'19 is assumed to be Spirostan-3-*O*-[*β*-d-Glucopyranosyl-(1 → 2)-[*β*-d-glucopyranosyl-(1 → 3)]-*β*-d-glucopyranoside]. Exclusion of the furostanol possibility; Furost-20(22)-ene-3,26-diol; (3*β*,5*β*,25R)-form, 3-O-[*β*-d-Glucopyranosyl-(1 → 2)-*β*-d-glucopyranoside], 26-*O*-*β*-d-glucopyranoside was based on the different mechanism of fragmentation of furostanols in positive mode, illustrated by the ion formation in MS1 which takes place by cleavage of the free and rather labile HO-C(22) group, which is absent in the spirostanol type, resulting in a prominent dehydrated pseudo-molecular ion, [(M-H_2_O) + H]^+^^54^.

Compounds P'16: Several 7 successive peaks were detected in the total ion chromatogram, in the region from retention time 6.2 to 6.27 min along with several successive scans, all having the same mass spectrum. All of these compounds showed a pseudomolecular ion [M + H]^+^ at m/z 741.4404, followed by a peak at m/z 579.3858, due to loss of one hexose unit and another peak at m/z 417.1661 due to loss of another hexose unit, yielding the aglycone fragment [M + H-162-162]^+^ of a spirostanol. All the compounds were identified as spirostanol dihexoside isomers, previously reported and recorded in the Dictionary of Natural products database as described in the first analysis. Compounds are identified as (3*β*,5*α*,25*R*)-form of Spirostan-3-*O*-[*β*-d-Galactopyranosyl-(1 → 4)-*β*-d-glucopyranoside], (3*β*,5*α*,25*R*)-form, (3*β*,5*β*,25*S*)-form and (3*β*,5*β*,25*R*)-form of Spirostan-3-*O*-[*β*-d-Glucopyranosyl-(1 → 2)-*β*-d-glucopyranoside].

Compound P'28, representing the major peak in the TIC, showed a pseudomolecular ion [M + Na]^+^ at m/z 438.2897 correspondings to [415 + Na] identified as spirostanol. Fragments ions at m/z 377.1358 and 253.1430 are characteristic steroidal saponin fragments due to ring cleavage^55^.

Compound P'25 was identified as hecogenin, an aglycone reported in many *Yucca* species. The compound showed a pseudo molecular ion [M + H]^+^ at m/z 431.314, followed by characteristic fragments at m/z 413.3, 395.29, and 299.24.

Compound P'23 showed a pseudo molecular ion at m/z 747.2970, followed by a peak at m/z 433.1765. This compound was identified as Yucca spirostanoside B2^53^.

Compound P'31 showed a pseudo molecular ion at m/z 417.3366 and was identified as Sarsasapogenin.

#### Identification of phenolic acids

Three phenolic acids were identified in the positive mode by comparison of their exact masses and fragmentation patterns.

Compound P'3 showed a pseudo molecular ion [M + Na]^+^ at m/z 527.1594, followed by two successive peaks at m/z 365.1031 and 203.0554, corresponding to [M + Na-162]^+^ and [M + Na-162-162]^+^, respectively, due to the loss of two hexose units, corresponding to [M + Na-162-162]^+^. The compound was identified as caffeoyl dihexoside.

Compounds P'4 and P'5 showed pseudo molecular ion peaks [M + Na]^+^ and [M + H]^+^ at m/z 193.0202 and 195.124, corresponding to that of gallic acid and ferulic acid, respectively^34^.

Compound P'6 showed a pseudomolecular ion peak [M + H]^+^ at m/z 149.0241, corresponding to that of cinnamic acid^35^.

#### Identification of flavonoids

Compound P'27 showed a pseudomolecular ion peak [M + H]^+^ at m/z 302.77, corresponding to quercetin. Characteristic fragmentation pattern of quercetin was observed as peaks at 273, 181, and 178.

Compound P'9 showed a pseudo molecular ion peak [M + H]^+^ at m/z 611.12, identified as hesperidin.

Compound P'12 showed a pseudo molecular ion peak [M + H]^+^ at m/z 625.167 and was identified as isorhamnetin-3-*O*-rurtinoside.

Compound P'22 showed a pseudo molecular ion peak [M + H]^+^ at m/z 581.1996, followed by a fragment at m/z 419.3506, corresponding to [M + H-162]^+^, due to loss of hexose unit, followed by the loss of a rhamnose unit, giving fragment ion [M + H-162-146]^+^ at m/z 273.2141, corresponding to the flavanone aglycone naringenin. This compound was identified as Naringin.

Compound P'20 showed a pseudo molecular ion peak [M + H]^+^ at m/z 286.88 and fragment ion at 147, was identified as a 4′,5-dihydroxy-7-methoxyflavone.

#### Identifications of compounds of steroidal nature

Compounds P'29, P'32, P'33, and P'34 were identified by the in-house database as phytol, diosgenin, cholesterol, and stigmastanol, respectively.

#### Identification of other compounds

Compounds P'7, P'8, P'11, and P'14 were identified by the in-house database as sweroside, 6-methylcoumarin, loliolide, and cinnamaldehyde, respectively.

The complexity of the extract was evidenced by a large number of detected peaks; 3706 in the positive mode and 5332 in the negative mode. In addition to the detection of successive resolved saponin peaks at very close retention times, with almost the same mass spectra. This confirms the presence of isomers with the same molecular weight, probably due to slight stereochemical differences, that lead to their separation on the liquid chromatography column and during mass detection successive cycles but could not aid their identification.

### LC–MS/MS comparison of the 4 plant extracts

The total ion chromatograms of the four investigated species; *Y. aloifolia* L., *Y.aloifolia variegata* L., *Y. filamentosa* L. and *Y. elephantipes* Regel. were superimposed to illustrate the differences between the different extracts(Table 7& FigureS2).

The most remarkable observation was the higher concentration of constituents in *Y.aloifolia variegata* L., proven by the higher intensity of most peaks compared to the same peaks in other extracts. This high concentration may explain the better biological activity of *Y.aloifolia variegata* L. which previously reported^19^. Where, *Y.aloifolia variegata* L. was evidenced cytotoxic against four types of cancer cell lines namely, lung cancer A549, liver cancer HEPG2, colon cancer Caco-2, and breast cancer. Hepatoprotective assay previously carried out^19^ on the extracts of the tested plants proved that *Y.aloifolia variegata* L. is the most potent one.

*Y. aloifolia* L. showed a similar total ion chromatogram to that of *Y.aloifolia variegata* L. in terms of compounds present at the same retention times and having the same fragmentation patterns. However, the intensities of all compounds were less than in *Y.aloifolia variegata* L.

*Y. filamentosa* L. showed a somewhat different profile. Saponin-rhamnosyl-hexosyl, hecogenin rhamnoside, spirostanol-dihexosides, and spirostan-diol-dihexosides were absent.

*Y. elephantipes* showed also a slightly different chromatogram. Sapon-rhamnosyl-hexosyl was not detected, in addition to the presence of spirostan-diol-monohexosides instead of spirostan-diol-dihexosides present in *Y.aloifolia variegata* L. and *Y.aloifolia* L.

These results imply that *Y.aloifolia variegata* L. extract was the richest extract, which was apparent in its superior biological activity.

## Methods

### Plant material

Samples of *Yucca aloifolia* L., *Yucca aloifolia variegata* L., *Yucca filamentosa* L., and *Yucca elephantipes* Regel were collected in January 2015 from Orman botanical garden, Cairo, Egypt. The four plants were identified by, Mrs. Therese Labib and Mr. Gamal El Kholy, Orman botanical garden, Cairo, Egypt. Voucher samples (2015-2-25A-D, respectively) were deposited at the Department of Pharmacognosy, Faculty of Pharmacy, Cairo University, Egypt.

*For phytochemical investigation*: Dried powdered leaves of the four plants were finely ground, each separately, macerated in 70% ethanol in a stoppered container and allowed to stand for two weeks with frequent agitation. The extracts were concentrated at 40 °C using a rotary evaporator. Hydroalcoholic fractionation was done to the most biologically active plant; which according to the biological investigation was Yucca aloifolia variegata. Fractionation was done using hexane, followed by chloroform, ethyl acetate, and finally *n*-butanol.

### Plant extracts

The air-dried powdered leaves of *Y. aloifolia variegata* (2 kg) were subjected to exhaustive cold maceration in 70% ethanol with frequent agitation. The extract was later collected and concentrated under vacuum at 40 °C. The concentration step was very tedious due to the constant frothing traced back to the presence of saponins. The residue left after evaporation of ethanol weighed 47.181 g.

#### Fractionation of the ethanolic extract

The dried ethanolic residue was suspended in the least amount of distilled water and then fractionated by shaking with n-hexane (5 × 250 ml), chloroform (7 × 250 ml), ethyl acetate (4 × 200 ml), and *n*-butanol saturated with water (7 × 250), successively. The separation was also very troublesome due to the difficulty in determining clear interphase between layers due to the surface-active activity of saponins. The extracting solvents, in each case, were removed under vacuum. The solvents in each case were evaporated to dryness under vacuum at a temperature not exceeding 40ºC, and the fractions weighed 6.17 g, 8.7 g, 5 g, and 14 g, respectively and the remaining aqueous fraction weighed 5 g.

### Chemicals

Authentic reference materials: Authentic fatty acids methyl ester standards for GC/MS analysis: The Supelco 37 component FAME Mix (Sigma-Aldrich) was used for the determination of present fatty acids in the saponifiable part of hexane fraction. Test solutions and solvents: Simple reagents such as acids (formic, glacial acetic acid, hydrochloric and sulfuric), alkalies (sodium hydroxide and potassium hydroxide), and alcoholic *α*-naphthol were obtained from faculty of pharmacy, Pharos University laboratories. All solvents used in this study (petroleum ether 60–80 °C, ethanol 95%, *n*-hexane, chloroform, dichloromethane, ethyl acetate, *n*-butanol, acetone) were purchased from El Gomhoria Company for Trading Pharmaceutical Chemical and Medical Appliances, Alexandria, Egypt.

### Materials for antimicrobial effect

Penicillin/streptomycin was purchased from Lonza bio Whittaker and B 4800 Verviers Belgium and used at a concentration of 100 units/ml and 100 µg/ml, respectively. Standard strains for antimicrobial activity screening: *Candida albicans* ATCC2091, *Escherischia coli* ATCC8739, *Pseudomonas aeruginosa* ATCC9027, *Staphylococcus aureus* ATCC6538, *Bacillus subtilis* ATCC19659.

### Apparatus

Gas chromatography-Mass spectrometer Device: The GC/MS analysis for both saponifiable and unsaponifiable fractions of the hexane extract was carried on Agilent GC–MS system, model 6890, fitted with Mass Selective Detector, in addition to a Thermo TR-FAME for fatty acid methyl ester analysis. The column is a 70% Cyanopropyl Polysilphenylene Siloxane (30 m, 0.25 ID, 1.4 μm film thickness) available at Central Laboratory Unit of the National Institute of Oceanography and Fisheries (NIOF), Alexandria. LC–MS/MS devices: First analysis: Analysis was performed using an Agilent 1200 HPLC column system composed of a quaternary pump with an online degasser, a thermostated column compartment, an autosampler, and Masshunter 1200 software, coupled to electrospray ionization (ESI) 6420 Triple Quad-LC/MS Agilent mass spectrometer. Second analysis: Analysis was performed using Exion nano-LC column (Sciex) with a cooled autosampler, a quaternary pump with an online degasser, a thermostated column compartment, and Analyst TF 1.7.1 software coupled to SCIEX electrospray ionization (ESI) TripleTOF 5600+ System for LC/MS–MS analysis of compounds at high resolution.

### Antimicrobial activity screening

The four plants were investigated for their antimicrobial activities against five standard microorganisms, including two gram-positive, two gram-negative, and one fungus.

*Sample preparation*: Each freeze-dried total extract was redissolved once in HPLC grade methanol and once in sterile water for injection. Each time, the redissolved extract was sonicated for 10 min followed by filteration through a 0.4 μm membrane filter.

Screening of antimicrobial activity was done using the Disc Diffusion Method^18^. Inhibition zones were measured in mm, for the extracts, the standard antibiotics, and the negative control. Experiments were carried out in triplicates. Results of screening of antimicrobial activity were compared with those obtained by standard antibiotic Ciprofloxacin and standard antifungal Clotrimazole. A negative control containing the solvent only was also included in the experiment. Microbial organisms used were: Standard strains of microorganisms used for antimicrobial activity screening: *Candida albicans* ATCC2091, *Escherischia coli* ATCC8739, *Pseudomonas aeruginosa* ATCC9027, *Staphyllococcus aureus* ATCC6538P, *Bacillus subtilis* ATCC19659.

### Gas chromatography-mass spectrometry (GC–MS)

#### Saponification of hexane fraction

An aliquot of the dried hexane extract (2 gm) was refluxed with 15 ml alcoholic potassium hydroxide (10%) for 5 h. The reaction mixture is then cooled and diluted with 20 ml distilled water. The unsaponifiable matter is then extracted with petroleum ether (3 × 20 ml) through shaking in separating funnel. The petroleum ether extracts are combined and washed with distilled water till free from alkalinity. The extract is then dried over anhydrous calcium chloride and filtered. The unsaponifiable fraction is finally dried under vacuum using a rotary evaporator, to be ready for GC/MS analysis.

The remaining aqueous mother liquor left after removal of petroleum ether was acidified with 10% HCl to liberate free fatty acids. The mixture is extracted with petroleum ether (3 × 20 ml), extracts are combined and dehydrated over anhydrous calcium chloride. Petroleum ether is evaporated under pressure till having a constant weight of the saponifiable matter.

#### Fatty acids methylation

Methylation of the free fatty acids from the saponifiable fraction was performed by refluxing the dry saponifiable residue with 50 ml absolute methanol and 3 ml concentrated sulfuric acid. Reflux was done in a water bath for 2 h at 100 °C. The reaction mixture is cooled and then extracted with petroleum ether (3 × 20 ml). The extract is then dried to obtain a dry residue of fatty acids methyl ester.

#### Conditions for GC/MS analysis of lipoidal constituents

The unsaponifiable fraction and the fatty acids methyl esters were subjected to GC/MS analysis under the following conditions; A capillary column packed with 70% Cyanopropyl Polysilphenylene Siloxane (30 m, 0.25 ID, 1.4 μm film thickness), Injector temperature: 300 °C, temperature transfer line: 350 °C, temperature programming: Initial temperature is 90 °C held for 2 min and then a gradual temperature increase at 3 °C/min till it reaches 350 °C with a hold time of 5 min., Carrier gas: He (1.5 ml/min), Sample injection volume: 1 μl, Ionization energy: 70 eV, Run time: 49 min. Identification of different constituents:

In the case of unsaponifiable constituents, identification was done by comparison of their retention times and relative retention time to 4-4(ethylcyclohexyl)-1-pentylcyclohexene and mass spectra to those indexed in the NIST library and the Wiley database. Only compounds of high matching probability were considered.

In the case of fatty acid methyl esters from the saponifiable fraction, identification was done by comparison of their retention times and relative to the retention time of palmitic acid also mass spectra to those of authentic fatty acid methyl esters co-injected with the sample.

### Conditions for LC–ESI–MS/MS analysis

*First analysis* LC–MS/MS ion trap (Institute of Marine Sciences, Alexandria University): HPLC separation was performed on XDB C18 column (50 mm × 2.1 mm, 1.8 μm, Agilent Company, USA). Mobile phase consisted of two solvents; solvent (A) acetonitrile and solvent (B) Deionized water with 1% formic acid. Gradient elution was performed at a flow rate of 0.8 ml/min at room temperature. Elution profile was isocratic from 0 to 4 min, (10% (A), 90% (B)), from 4 to 8 min, (20% (A), 80% (B)), from 8 to 12 min, (30% (A), 70% (B)), from 12 to 16 min, (40% (A), 60% (B)), from 16 to 20 min, isocratic (50% (A), 50% (B)), from 20 to 24 min, (60% (A), 40% (B)), from 24 to 28 min, (70% (A), 30% (B)), from 28 to 32 min, (80% (A), 20% (B)), from 32 to 36 min, (90% (A), 10% (B)), from 36 to 45 min, (100% (A), 0% (B)).

*Mass spectrometric conditions*: The ionization parameters were as follows: Positive ion mode, capillary voltage 4000 V, endplate voltage -500 V; nitrogen was used as nebulizing gas at 35.0 p.s.i. Mass analyzer scanned m/z range from 120 to 1000 amu. The fragmentation amplitude was set to 135 eV. MS2 data were acquired in positive ion mode.

*Second analysis* Triple TOF 5600+ (57357 hospitals): HPLC separation was performed on (Waters) reversed-phase Exion Xbridge C18 column (2.1 × 50 mm, 3.5 µm) preceded by a (Phenomenex) precolumn, in-Line filter disks (0.5 µm × 3.0 mm). The mobile phase consisted of two solvents, for each mode, solvent (A) Deionized water containing 0.1% formic acid, solvent (B) 5 mM ammonium formate buffer (pH 8) containing 1% methanol, and solvent (C) 100% acetonitrile. For the negative ion mode, solvents (A) and (C) were used while for the positive ion mode, solvents (B) and (C) were used. 20 µl stock (50/1000 µl) was diluted with 1000 µl reconstitution solvent. Finally, the injected concentration was 1 µg/µl. Gradient Elution was performed at a flow rate of 0.3 ml/min at 40 °C, where from 0 to 1 min, isocratic (90% (A) or (B), 10% (C)), from 1 to 25 min, linear gradient from 90 to 10% (A) or (B), 10% to 90% (C). From 25.01 to 28 min, elution was isocratic (90% (A) or (B), 10% (C)). Solvent (A) was used for negative ion mode only, while Solvent (B) was used for positive ion mode only.

*Mass spectrometric conditions*: The ionization parameters were as follows: negative ion mode; duration of the run was 28 min including 2584 cycles, 0.6502 secs each. The range of mass detected was from 50 to 1000 Da. For MS1 acquisition, nebulizer gas GS1: nitrogen, drying gas GS2: nitrogen and curtain gas CUS flow rates were 45, 45, and 25 psi, respectively. The temperature was 500 °C and the ion spray voltage was − 4500 V. For MS2 acquisition, a declustering potential of 80 V, collision energy CE of 35 V, and collision energy spread CES of 20 V were applied, respectively. Switch criteria were as follows; former ions were excluded after 3 repeats, former target ions after 3 s, and exclusion of isotopes within 2 Da. The maximum number of candidate ions to monitor per cycle was 15. Positive ion mode; has the same parameters but with an ion spray voltage of 4500 V.

## Conclusion

The use of the total extract of *Yucca aloifolia variegata* L. is recommended due to the almost similar composition of different fractions. The chemical composition of the alcoholic extract of *Y. aloifolia variegata* L. was investigated using MS-techniques at different voltages. Analysis proved the main constituents of *Y. aloifolia* variegata L. were flavonoids, phenolic acids, and saponins. Variations in the results of LC–MS analyses are mainly due to differences in techniques used. The lower the collision energy, the better was the identification of compounds. Flavonoids and phenolics are better detected in the negative mode of ESI–MS/MS. Saponins are better detected in the positive mode of ESI–MS/MS.

## Supplementary information

Supplementary Legends.

Supplementary Figure S1.

Supplementary Figure S2.

Supplementary Figure S3.

Supplementary Figure S4.

Supplementary Figure S5.

Supplementary Figure S6.
