# Supplementary material for: LC–MS/MS and GC–MS profiling as well as the antimicrobial effect of leaves of selected Yucca species introduced to Egypt
Source: Sci Rep. 2020 Oct 20;10:17778. doi: 10.1038/s41598-020-74440-y (PMC7575531; doi:10.1038/s41598-020-74440-y)
Supplement: Supplementary file 1 — Supplementary Legends. [file 41598_2020_74440_MOESM1_ESM.docx]

**Figure S1 a**: GC-MS analysis of the saponifible fraction of *Y. aloifolia variegata* L. extract. **b**: Relative percentage of different components in the saponifiable fraction **c**: GC-MS analysis of the unsaponifible fraction of *Y. aloifolia variegata* L. extract **d**: Relative percentage of different components in the saponifiable fraction.

**Figure S2**: LC-MS/MS total ion chromatogram(TIC) of the 4 Yucca plants extracts; *Y. aloifolia* variegata L. (blue), *Y .aloifolia* (red), *Y. filamentosa* (green), *Y. elephantipes* (magenta).

**Figure S3**: LC-MS/MS total ion chromatogram (TIC) **a**: in positive ion mode 135eV. **b**: in negative ion mode 35 eV C: in positive ion mode 35 eV of the total alcoholic extract of *Y. aloifolia variegata* L. leaves.

**Figure S4**: Structures of compounds identified through LC-MS/MS in postive ESI mode of ethanol extract of *Y. aloifolia* variegata L , Y1= Spirostan-3-ol-3-O-[β-D-galactopyranosyl-(1→4)-β-D-glucopyranoside, Y2= Gallic acid, Y3= Spirostan-diol- rhamnosyl hexosyl, Y4= Hecogenin-rhamnoside , Y5=Spirostan-3-ol-3-O-[β-D-glucopyranosyl-(1→2)-β-D-glucopyranoside, Y9, =Spiostan-diol-dihexoside,Y12 =Chlorogenic acid, Y13= rutin/ hesperidin Y14= cinnamic acid .

**Figure S5**: Structure of compounds identified through LC-MS/MS in negative mode ,P1= Malic acid, P2= Hesperidn, P3= caffeic acid, P4 =3,5,7-trihydroxy-4'-methoxyflavone, P8= Kaempferol-3-O-Glucuronid,P6=ferulic or isoferulic acid,P9 Quercetin-O-hexoside,P15= Neoeriocitrin, P= 19 Kaempferol dirhamnoside, P 22 Isorhamnetin-3-O-rutinosideP 25 Spirostanol-3-O-dihexoside, P 26 Hecogenin, P33= Quercetin , P37 Procyanidin , P40 Spirostanol, P= 41 Linoleic acid.

**Figure S6:**Structures of identified compounds through LC-MS in (+)ESI-MS spectrum of ethanol extract of ethanolic extract of Y. aloifolia variegate, compound P'3 caffeoyl dihexoside P'4 Gallic acid, P'6 cinnamic acid , P'10 Spirostan-3,12-diol-3-O-glucopyranosyl-(1→2)-glucopyranosyl, P'15 spirostanol dihexosideP'16, P'19 spirostanol trihexoside, P'22 Naringin P'28 Spirostanol, P'30 Yucca spirostanoside B2.
