# Supplementary figures and images for: LC–MS/MS and GC–MS profiling as well as the antimicrobial effect of leaves of selected Yucca species introduced to Egypt
Source: Sci Rep. 2020 Oct 20;10:17778. doi: 10.1038/s41598-020-74440-y (PMC7575531; doi:10.1038/s41598-020-74440-y)

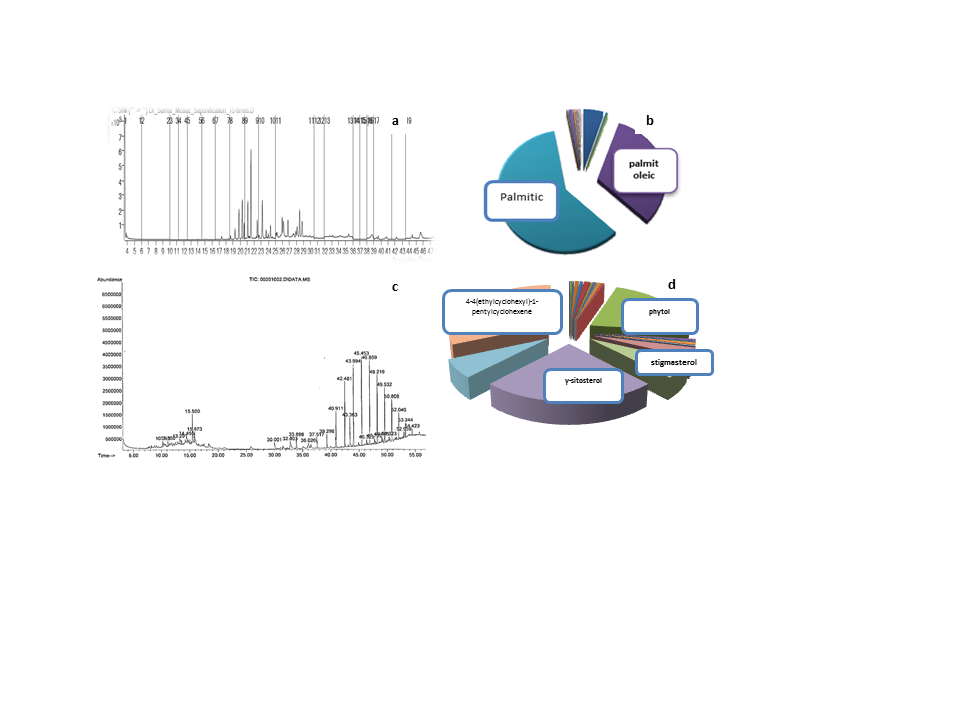

Supplement: Supplementary file 2 — Supplementary Figure S1. [file 41598_2020_74440_MOESM2_ESM.tif]

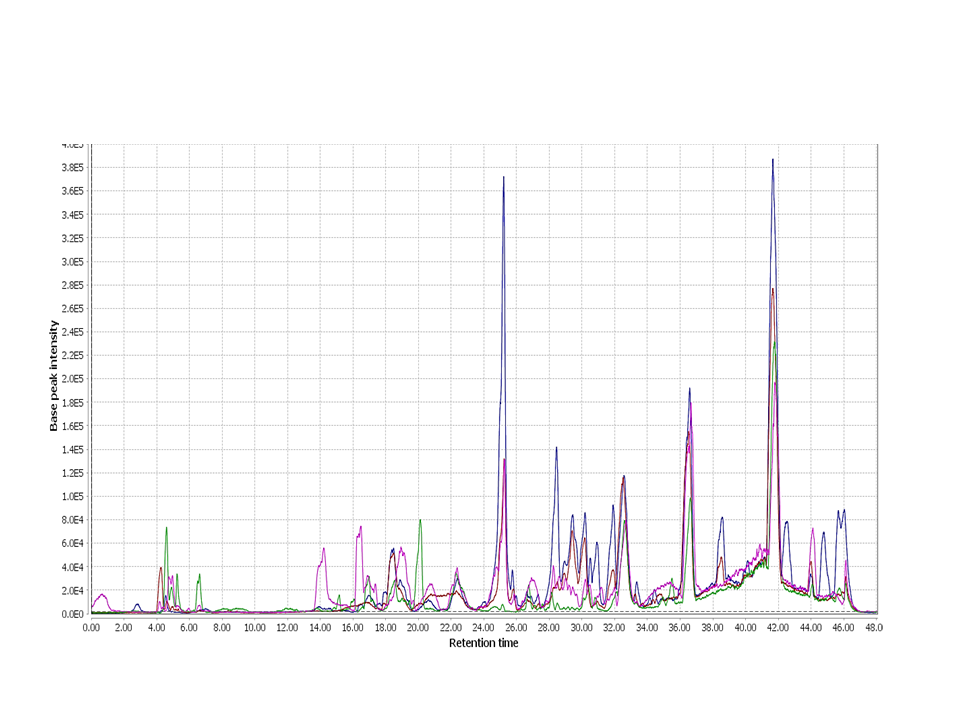

Supplement: Supplementary file 3 — Supplementary Figure S2. [file 41598_2020_74440_MOESM3_ESM.tif]

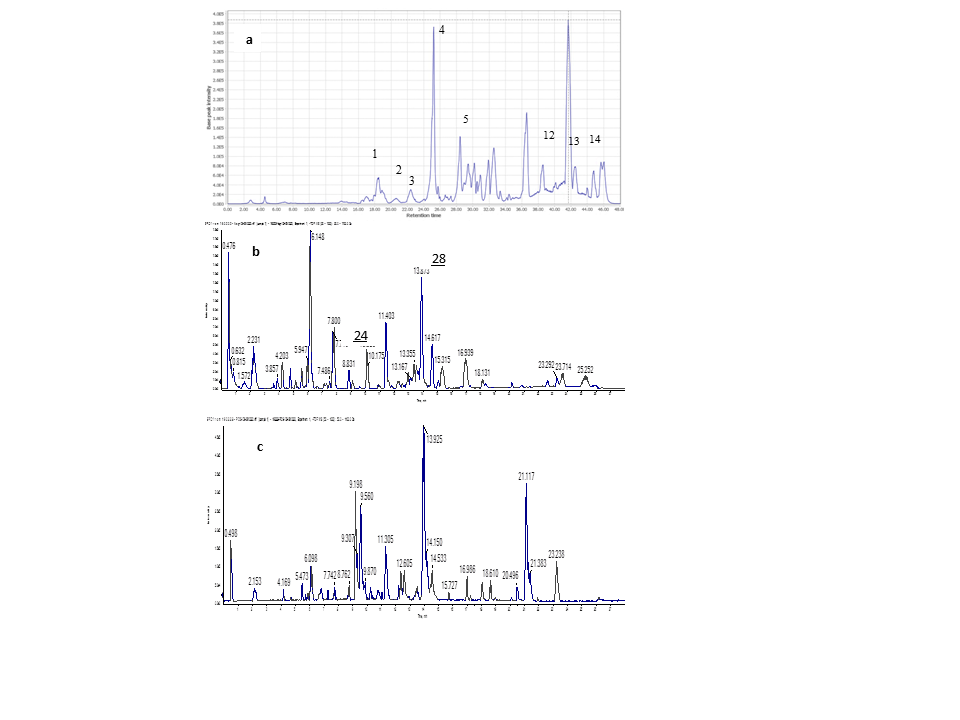

Supplement: Supplementary file 4 — Supplementary Figure S3. [file 41598_2020_74440_MOESM4_ESM.tif]

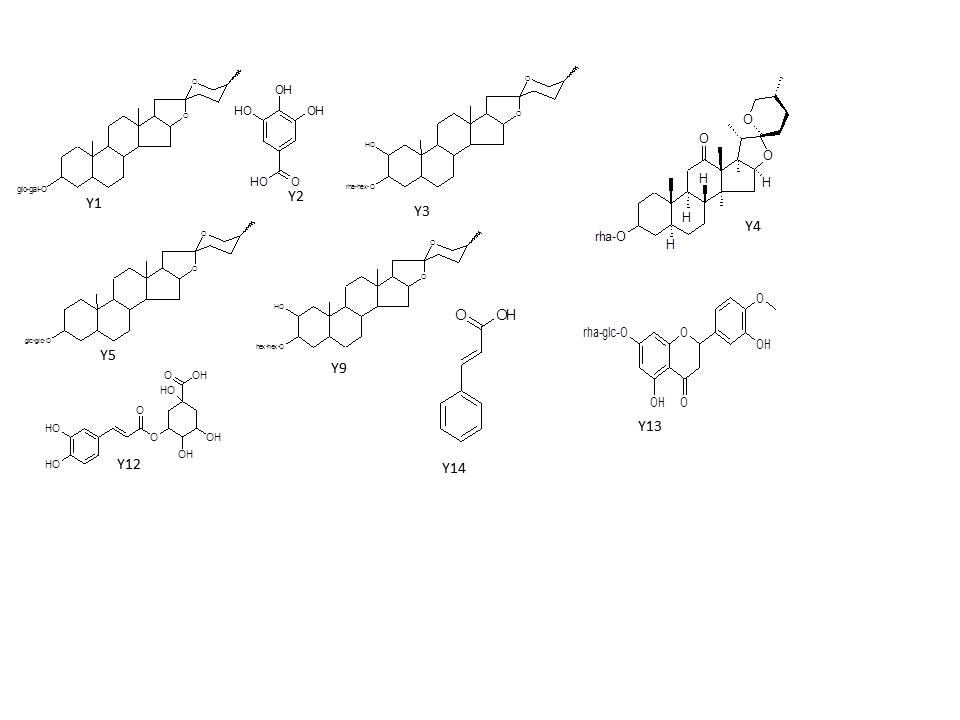

Supplement: Supplementary file 5 — Supplementary Figure S4. [file 41598_2020_74440_MOESM5_ESM.jpg]

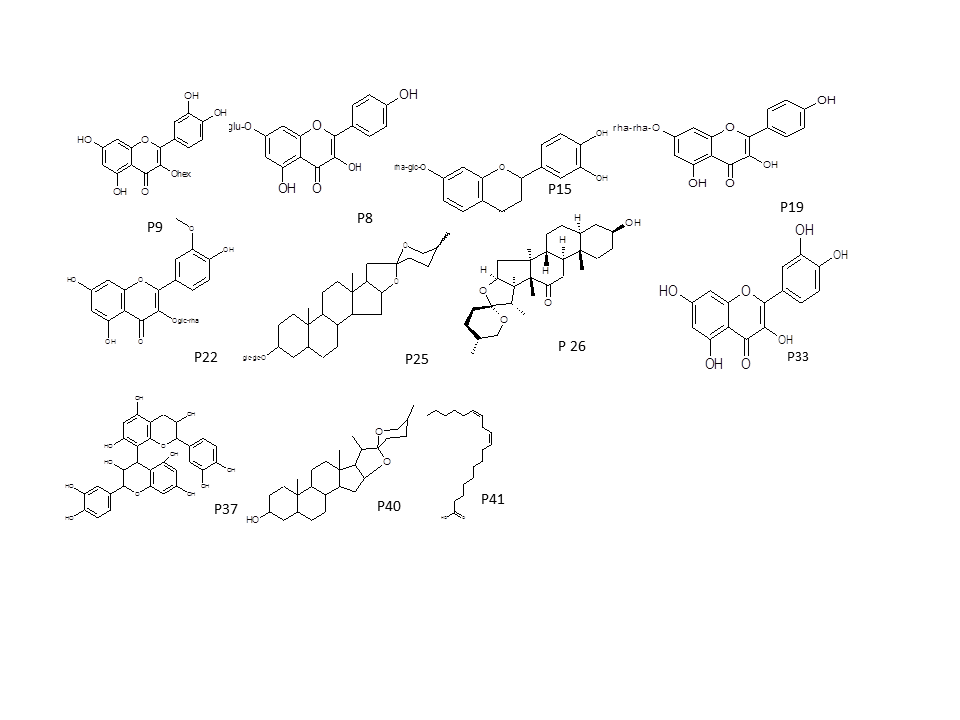

Supplement: Supplementary file 6 — Supplementary Figure S5. [file 41598_2020_74440_MOESM6_ESM.tif]

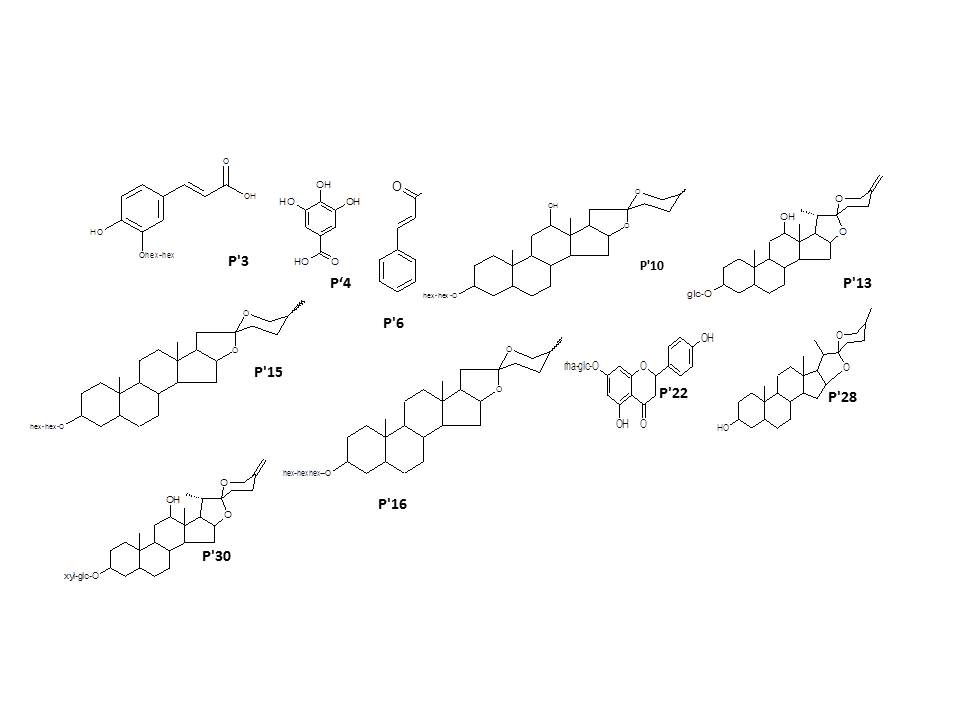

Supplement: Supplementary file 7 — Supplementary Figure S6. [file 41598_2020_74440_MOESM7_ESM.tif]
